# Ausência de Descenso da Pressão Arterial Detectada pela Monitorização Ambulatorial da Pressão Arterial em Pacientes com Doença de Chagas Aguda Transmitida por Via Oral

**DOI:** 10.36660/abc.20190143

**Published:** 2020-05-12

**Authors:** Dilma do S. M. de Souza, Céres Larissa Barbosa de Oliveira, Brenda Gonçalves Maciel, Maria Tereza Figueiredo, Henrique Tria Bianco, Francisco A. H. Fonseca, Maria Cristina Izar, Rui M. S. Póvoa

**Affiliations:** 1 Universidade Federal do Pará Belém PA Brasil Universidade Federal do Pará, Belém, PA - Brasil; 2 Universidade Federal de São Paulo Escola Paulista de Medicina São Paulo SP Brasil Universidade Federal de São Paulo – Escola Paulista de Medicina, São Paulo, SP - Brasil

**Keywords:** Doença de Chagas/fisiopatologia, Pressão Arterial/fisiologia, Sistema Nervoso Autônomo/fisiologia, Monitoração Ambulatorial da Pressão Arterial/métodos, Hipertensão

## Abstract

**Fundamento:**

O acometimento do sistema nervoso autônomo é um dos mecanismos propostos para explicar a progressão da lesão miocárdica na doença de Chagas. Evidências indicam alterações do sistema nervoso simpático e parassimpático desde a fase aguda, e estudos são necessários para se entender os aspectos fisiopatológicos e o valor prognóstico dessas alterações.

**Objetivo:**

Analisar o comportamento da pressão arterial pela monitorização ambulatorial da pressão arterial (MAPA) em pacientes normotensos com doença de Chagas aguda (DCA) sem envolvimento cardíaco aparente, e a influência da infecção no descenso fisiológico do sono.

**Métodos:**

Foi realizado a MAPA em 54 pacientes com DCA e utilizado um grupo controle de 54 indivíduos normotensos, pareados para idade e sexo. O nível de significância adotado foi para um erro tipo I (alfa) de 5%.

**Resultados:**

Em um total de 54 pacientes com DCA ocorreu ausência de descenso sistólico do sono em 74,0%*, ausência de descenso diastólico do sono em 53,7%*, e ausência de descenso sistólico e diastólico do sono (51,8%)*, (*p<0,05). Em 12,9% ocorreu ascensão sistólica da pressão no sono e em 18,5% ascensão diastólica (p<0,05).

**Conclusão:**

Em pacientes com Doença de Chagas aguda, houve ausência significativa do descenso fisiológico da pressão arterial durante o sono, tanto da pressão arterial sistólica quanto a diastólica, e alguns pacientes apresentaram ascensão noturna desses parâmetros. Esses achados sugerem alterações autonômicas na doença de Chagas desde a fase aguda. (Arq Bras Cardiol. 2020; 114(4):711-715)

## Introdução

A doença de Chagas é uma zoonose causada pelo protozoário hemoflagelado *Trypanosoma cruzi* ( *T. cruzi* ). A doença é endêmica em 21 países latino-americanos e gera alto impacto social dada sua morbimortalidade elevada. De acordo com a Organização Mundial da Saúde (OMS), estima-se que haja de 6 a 7 milhões de pessoas infectadas em todo mundo, grande parcela delas na América Latina.^1^ A forma clássica de transmissão vetorial ao homem vem diminuindo em zonas endêmicas na América Latina devido às iniciativas de controle da infecção. Porém, o desmatamento intenso na região Amazônica, associado às migrações populacionais provocam mudanças do cenário epidemiológico, ocorrendo aumento expressivo da transmissão oral.^2^

Na transmissão por via oral, a apresentação clínica mais frequente da fase aguda inclui síndrome febril prolongada, frequentemente relacionada com surtos de microepidemia familiar, e diversos sintomas inespecíficos, característicos da forma vetorial, porém com maior morbidade e mortalidade.^3 - 5^ Na forma crônica da doença de Chagas encontramos, na maioria dos pacientes, importantes alterações no sistema autônomo, com aumento da atividade simpática e diminuição da parassimpática; entretanto, à luz do atual conhecimento, são desconhecidas as possíveis alterações autonômicas durante o acometimento agudo. Muitos pacientes apresentam alterações na pressão arterial (PA), e a monitorização ambulatorial da pressão arterial (MAPA) revela alterações na pressão arterial durante o sono, principalmente no descenso. Nos pacientes não chagásicos este evento tem sido interpretado como sinal de disautonomia e possível preditor de risco cardiovascular.^6^ Em vista do declínio fisiológico da PA durante o sono estar relacionado com a regulação autonômica, é importante a MAPA na doença de Chagas aguda (DCA) para uma melhor compreensão do comportamento pressórico, sobretudo no período do sono. Portanto, o objetivo do presente estudo foi avaliar o comportamento da PA em pacientes com DCA, por meio da MAPA.

## Métodos

Este foi um estudo de centro único realizado em hospital universitário. Foi realizada a MAPA em 54 pacientes (amostragem por conveniência) com DCA por contaminação oral, atendidos em ambulatório de referência em doenças infecciosas e parasitárias e um grupo controle com 54 participantes saudáveis, pareados por idade e gênero. O objetivo da utilização de um grupo controle neste estudo foi avaliar a prevalência da ausência do descenso noturno na população sem comorbidades, tendo em vista que essa variável não foi estudada na população brasileira. Os participantes eram saudáveis e sem nenhuma queixa ou história de qualquer doença, com exame clínico normal e sem uso de nenhuma medicação no período basal. Todos os participantes, ou representantes legais assinaram termo de consentimento informado.

Foi utilizado para a monitorização da pressão arterial o aparelho da marca Dyna-MAPA^®^. Os critérios de inclusão foram: pacientes ambulatoriais com diagnóstico de DCA utilizando-se o critério clínico, parasitológico e/ou sorológico positivo somado ao vínculo epidemiológico de acordo com protocolo do ministério da saúde, disponível em: http://portalms.saude.gov.br/saude-de-a-z/doenca-de-chagas. Os critérios de exclusão foram: presença de diabetes *mellitus* , doenças neurológicas, hipertensão arterial, doença cardiovascular prévia, outras infecções em curso, distúrbio hematológicos como anemia, uso de drogas ilícitas, doenças com comprometimento da função renal, disfunções de tireoide, gravidez, alcoolismo ou outras alterações sistêmicas de relevância. Para o grupo controle foram analisadas as MAPAs de indivíduos normotensos. O exame foi solicitado não por suspeita de hipertensão, e sim para avaliação periódica de saúde. Não foram incluídos pacientes com qualquer tipo de doença cardíaca.

Foram avaliadas as seguintes variáveis pela MAPA: pressão arterial sistólica (PAS) e pressão arterial diastólica (PAD) no período de 24 horas, na vigília e no período do sono, e descenso do sono. O descenso fisiológico para a PAS e PAD foi considerado quando houvesse redução ≥ 10% na média dos valores registrados no período de vigília. Considerou-se como período de vigília na MAPA o período entre 8 horas às 20 horas e período de sono entre 20 horas e 8 horas do dia seguinte, de acordo com as recomendações das Diretrizes Brasileiras de MAPA e Monitorização Residencial da Pressão Arterial (MRPA III) de 2011.^7^

Este estudo foi aprovado pelo comitê de ética do Hospital Universitário João de Barros Barreto, sob Nº CAAE 01278918.4.00000017, e foi conduzido atendendo a resolução 466/12 e de acordo com os princípios éticos da Declaração de Helsinque.

### Análise estatística

Na análise estatística foi utilizado o teste do qui-quadrado para comparação dos indivíduos que não apresentaram descenso noturno da PA entre o grupo de pacientes *versus* grupo controle. Considerou-se o valor de p < 0,05 como estatisticamente significativo. Variáveis categóricas foram apresentadas como frequências, número absoluto e porcentagens. Variáveis com distribuição normal foram apresentadas como média e desvio padrão. Utilizaram-se o teste de Kolmogorov-Smirnov e a inspeção de normalidade por histograma, assim como a curtose e nível de assimetria. A análise estatística foi realizada com o SPSS 23.0 para Windows (IBM Corp. Lançado em 2015, IBM SPSS Statistics para Windows, Versão 23.0, Armonk, NY: IBM Corp).

## Resultados

No total de 54 pacientes com infecção aguda por *T. cruzi* , a idade média foi de 36,2 ±10,4 anos, onde 30 eram mulheres (idade média 34,7 ± 19,0 anos) e 24 homens (idade média 38,3 ± 19,7 anos).

A MAPA revelou que 40 pacientes (74,0%) apresentavam ausência de descenso sistólico do sono, sendo que 29 (53,7%) não apresentavam descenso diastólico. A ausência concomitante de descenso sistólico e diastólico ocorreu em 29 pacientes (53,7%); 7 (12,9%) apresentaram ascensão noturna da PAS e 10 (18,5%) pacientes da PAD.

Como pode ser visto na Tabela 1 , não foram identificadas diferenças estatisticamente significativas para as médias das pressões sistólicas e diastólicas nas 24 h, na vigília e no sono dos pacientes com DCA e do grupo controle. Na Tabela 2 , encontramos diferenças significativas quando comparamos o grupo de pacientes ao grupo controle, notadamente na variável descenso sistólico e diastólico do sono, em ambos os sexos.

**Tabela 1:** – Pressões arteriais médias nas 24h, na vigília e durante o sono de 54 pacientes normotensos com doença de Chagas aguda e 54 pacientes normotensos sem doença de Chagas (controle)

	Controle			DCA		

	24h	vigília	sono	24h	vigília	sono
**Média**	114,1±10,3	117,3±10,4	100,9±10,0	111,0±10,6	112,7±10,5	105,1±11,7
**PAS**						
**mmHg**						
**Média**	68,9±7,6	71,3±8,1	59,2±7,5	66,9±7,0	68,3±7,2	62,2±8,1
**PAD**						
**mmHg**						

*Dados são expressos como média e desvio padrão. PAS: pressão arterial sistólica; PAD: pressão arterial diastólica; DCA: doença de Chagas aguda.*

**Tabela 2:** – Número de pacientes com alterações na monitorização ambulatorial da pressão arterial (MAPA) no grupo controle e no grupo com doença de Chagas aguda

	Controle			Doença de Chagas aguda		

	Mulheres (n=30)	Homens (n=24)	Total (n=54)	Mulheres (n=30)	Homens (n=24)	Total (n=54)
**Ausência de descenso sistólico do sono**	5	4	9 (16,6%)	20	20	40 (74,0%)*
**Ausência de descenso diastólico do sono**	4	3	7 (12,9%)	9	20	29 (53,7%)*
**Ausência de descenso sistólico e diastólico do sono**	4	3	7 (12,9%)	16	12	28 (51,8%)*
**Ascensão sistólica da pressão no sono**	0	1	1 (1,8%)	5	2	7 (12,9%)*
**Ascensão diastólica da pressão no sono**	0	1	1 (1,8%)	5	5	10 (18,5%)*

Dados apresentados como números absolutos e percentuais. Teste do qui-quadrado para comparações entre os grupos doença de Chagas aguda e controle, sendo consideradas diferenças significativas quando p<5% (*).

## Discussão

Na fase crônica da doença de Chagas, as alterações do sistema nervoso autônomo são bem conhecidas, caracterizadas por perda neuronal e lesão do sistema parassimpático, com consequente aumento da atividade simpática.^8^

Estudos realizados em pacientes com a forma indeterminada da doença de Chagas demonstraram predominância de atividade parassimpática, a qual foi correlacionada com disfunção autonômica.^9^ Resultados de um interessante estudo apontaram para a existência de uma relação entre as alterações na modulação autonômica e a função endotelial em pacientes com a DCA.^10^ Lesões do sistema nervoso central foram observadas em estudos anatomopatológicos de paciente com DCA, sendo descritas lesões sistêmicas e à distância ocasionada pelo parasita em células ganglionares.^11 , 12^ Em todas as fases da doença de Chagas pode-se demonstrar o acometimento do sistema nervoso autônomo, e as alterações do controle autonômico parassimpático não foram correlacionadas com sintomas cardiovasculares por testes funcionais em humanos.^13^ A perda do controle autonômico na cardiopatia chagásica crônica foi descrita em estudo caso-controle que avaliou a correlação entre inervação simpática, alterações de perfusão e anormalidade de parede ventricular, mostrando que o comprometimento da função simpática cardíaca ocorre em fase precoce no curso evolutivo da cardiopatia chagásica e se associa com a progressão da disfunção ventricular.^14^ Porém, na fase aguda, são escassos os estudos relacionados com a função autonômica. As evidências demonstram acometimento do sistema nervoso autônomo principalmente do parassimpático logo após a infecção inicial, ou seja, na fase indeterminada da doença de Chagas.^15^

A variação fisiológica da pressão arterial tem característica circadiana, flutuando ao longo das 24 horas, mas com queda durante o sono. Essa queda, detectada pela MAPA, normalmente excede 10% da PA na vigília, e é observada em cerca de 95% dos indivíduos normotensos.^16^ Durante o sono, ocorrem mudanças específicas das funções autonômicas e endócrinas, com redução da atividade simpática e um predomínio da atividade parassimpática, proporcionando, assim, a queda pressórica fisiológica.^17 , 18^ As observações na prática clínica que muitos pacientes com DCA que realizaram a MAPA por qualquer motivo não apresentavam descenso da pressão durante o sono motivaram a realização de um estudo sistematizado para avaliar o comportamento da PA na MAPA. Foram incluídos no grupo controle somente indivíduos sadios do ponto de vista cardiovascular, sem história prévia de hipertensão, diabetes ou doenças cardiovasculares. Todos eram normotensos na avaliação de consultório (PA<140/90 mmHg, médias das duas últimas medidas). Os pacientes estavam somente em uso de benzonidazol, antiparasitário administrado na forma oral, usado de forma específica contra o *T. cruzi* o protozoário causador da Doença de Chagas, para o tratamento etiológico da DCA. Observou-se ausência do descenso do sono em uma proporção muito alta, em mais da metade dos pacientes com DCA, e ascensão noturna da pressão em taxa significativa de pacientes (12,9% para a sistólica e 18,5% para a diastólica).

Os aspectos neuro-humorais da fase aguda ainda são pouco conhecidos, notadamente devido às características epidemiológicas e as dificuldades do diagnóstico. Entretanto, atualmente, com a mudança do perfil da doença, e o aumento dos casos onde a contaminação ocorreu por via oral, com carga parasitária mais intensa, observamos um estado clínico mais exuberante.^19^ Esta ausência do descenso do sono, observada na fase aguda, pode ser reflexo de algum distúrbio no sistema nervoso autônomo. Uma limitação deste estudo foi não ter realizado a variabilidade RR, pois pretendíamos somente avaliar o comportamento da pressão nas 24h.

## Conclusões

Os resultados deste estudo sugerem que a MAPA pode ser uma ferramenta útil para detecção precoce de alterações autonômicas na fase inicial da doença de Chagas. Por se tratar de um estudo descritivo de pacientes na fase aguda da doença, não é possível entender o real significado dessas alterações, pois a ausência de descenso ainda é controversa quanto à reprodutibilidade e repercussões clínicas em longo prazo.
