# Supplementary material for: Ausência de Descenso da Pressão Arterial Detectada pela Monitorização Ambulatorial da Pressão Arterial em Pacientes com Doença de Chagas Aguda Transmitida por Via Oral
Source: Arq Bras Cardiol. 2020 May 12;114(4):711–5. [Article in Portuguese] doi: 10.36660/abc.20190143 (PMC9744338; doi:10.36660/abc.20190143)
Supplement: Translation

# Translation: Absence of Nocturnal Fall in Blood Pressure Detected by Ambulatory Blood Pressure Monitoring in Acute Chagas Disease Patients with Oral Infection

**Authors:** Dilma do S. M. de Souza, Céres Larissa Barbosa de Oliveira, Brenda Gonçalves Maciel, Maria Tereza Figueiredo, Henrique Tria Bianco, Francisco A. H. Fonseca, Maria Cristina Izar, Rui M. S. Póvoa

**Affiliations:** 1 Brazil Universidade Federal do Pará, Belém, PA - Brazil; 2 Brazil Universidade Federal de São Paulo – Escola Paulista de Medicina, São Paulo, SP - Brazil

**Keywords:** Chagas Disease/physiopathology, Blood Pressure/physiology, Autonomic Nervous System/physiology, Blood Pressure Monitoring, Ambulatory/methods, Hypertension

## Abstract

**Background:**

The involvement of the autonomic nervous system is one of the mechanisms proposed to explain the progression of myocardial lesion in Chagas disease. Evidences have shown changes in sympathetic and parasympathetic nervous system since the acute phase of the disease, and studies to clarify the pathophysiological and prognostic value of these changes are needed.

**Objetives:**

To assess blood pressure profile by ambulatory blood pressure monitoring (ABPM) in normotensive patients with acute Chagas disease (ACD) without apparent cardiac damage, and the influence of the infection on nocturnal blood pressure fall.

**Methods:**

ABPM was performed with 54 patients with ACD and a control group composed of 54 age- and sex-matched normotensive individuals. The alpha level of significance (type I error rate) was set at 5%.

**Results:**

In the total of 54 patients, 74.0% did not show nocturnal fall in systolic blood pressure, 53.7% did not show nocturnal fall in diastolic blood pressure, and lack of both nocturnal fall in SBP and DBP was observed in 51.8% (*p<0.05). In 12.9% of patients, there was an increase in SBP and in 18.5% increase in DBP (p<0.05).

**Conclusions:**

In patients with acute Chagas disease, a significant absence of the physiological fall in both systolic and diastolic blood pressure was observed during sleep, and some of the patients showed nocturnal increase in these parameters. These findings suggest autonomic changes in the acute phase of Chagas disease. (Arq Bras Cardiol. 2020; 114(4):711-715)

## Introduction

Chagas disease is a zoonosis caused by the flagellate protozoan *Trypanosoma cruzi* ( *T. cruzi* ) that feeds primarily on blood. The disease is endemic in 21 countries in Latin America, with an important social impact due to its high morbidity and mortality. According to the World Health Organization (WHO), it is estimated that 6-7 million people are infected, most of them in Latin America.^1^ The classical form of transmission – vector transmission – has been decreasing in endemic areas in Latin America thanks to infection control initiatives. However, intense deforestation in the Amazon region, in addition to migration of people, has changed the epidemiological scenario, with an expressive increase in oral transmission.^2^

The most common clinical presentation in the acute phase of the orally transmitted disease includes prolonged fever syndrome, usually associated with familial microepidemics, and several unspecific symptoms characteristic of vector transmission of Chagas disease, but with higher morbidity and mortality.^3 - 5^ In the chronic form of the disease, important changes in the autonomic system are observed, with increased sympathetic activity and decreased parasympathetic activity. However, autonomic changes in the acute phase of the disease are not known so far. Many patients have altered blood pressure (BP) and abnormal ambulatory blood pressure monitoring (ABPM) measures, mainly related to nocturnal BP fall. In non-chagasic patients, such event has been regarded as a sign of dysautonomia and possible predictor of cardiovascular risk.^6^ In light of the physiological decline in nocturnal BP, an ABPM in acute Chagas disease (ACD) is advisable, aiming at a better understanding of BP behavior, especially during sleep. Therefore, the aim of the present study was to assess BP behavior in patients with ACD using ABPM.

## Methods

This was a single-center study conducted in a university hospital. ABPM was performed in 54 patients (convenience sample) with orally transmitted ACD, seen in an outpatient clinic of infectious and parasitic diseases and 54 age- and sex- matched healthy controls. This control group was used aiming at evaluating the prevalence of lack of nocturnal BP fall in individuals without comorbidities, since this variable has not been investigated in the Brazilian population. The healthy controls had no complaints or history of any disease, had a normal clinical examination and were not taking any medication at baseline. All participants or their legal representatives signed an informed consent form.

The ABPM was carried out using a Dyna-MAPA^®^device. Inclusion criteria were patients attending the outpatient clinic with a diagnosis of ACD confirmed by a positive parasitological and/or serological test, in addition to meeting the epidemiological criteria established by the Brazilian Ministry of Health’s protocol, available at: http://portalms.saude.gov.br/saude-de-a-z/doenca-de-chagas. Exclusion criteria were presence of diabetes mellitus, neurological diseases, arterial hypertension, cardiovascular disease, ongoing infection, hematological disease such as anemia, conditions that may affect renal function, thyroid disease or other important systemic changes, use of illicit drugs, pregnancy and alcoholism.

In the control group, ABPM was performed with normotensive individuals. The test was ordered as a routine test (health check-up) rather than for suspected hypertension. Individuals with any type of cardiac disease were not included.

The following ABPM parameters were assessed: 24-hour systolic (SBP) and diastolic BP (SBP), BP during sleep and awake states, and BP fall during sleep. Physiological fall in SBP and DBP was considered as a reduction ≥ 10% in mean BP registered during sleep. The awake period was considered the period from 8 to 20 o’clock, whereas the sleep-period time from 20 to 8 o’clock on the day after, following the 2011 Brazilian Guidelines on ABPM and home blood pressure monitoring.^7^

The study was approved by the ethics committee of the Hospital Universitário João de Barros Barreto (CAAE 01278918.4.00000017).

### Statistical analysis

The chi-square test was used to compare individuals that did not show a nocturnal BP fall between patients and control group. A p<0.05 was set as statistically significant. Categorical variables were presented as frequency, absolute numbers and percentage. Normally distributed variables were presented as mean and standard deviation. The Kolmogorov-Smirnov test and the histogram-normality test were used, and measurement of asymmetry and kurtosis was performed. The SPSS 23.0 software for Windows (IBM SPSS *Statistics* para Windows version 23.0, launched in 2015, Armonk, NY: IBM Corp).

## Results

In the total of 54 patients with acute infection with *T. cruzi* , mean age was 36.2 ±10.4 years, 30 were women (mean age 34.7 ± 19.0 years) and 24 men (mean age 38.3 ± 19.7 years).

The ABPM showed that 40 patients (74.0%) did not show nocturnal fall in SBP, and 29 (53.7%) did not show nocturnal fall in DBP. This occurred concomitantly in 29 (53.7%) patients; seven (12.9%) showed nocturnal increase in SBP and 10 (18.5%) in DBP.

No statistically significant difference was found in the mean 24-hour SBP and mean 24-hour DBP during sleep and awake states between patients with ACD and control group ( Table 1 ). Significant differences were found between the groups for nocturnal fall in SBP and DBP in both sexes.

**Table 1:** – Mean values of 24-hour blood pressure during sleep and awake states in 54 normotensive patients with acute Chagas disease and 54 normotensive individuals without Chagas disease (controls)

	Controls			Acute Chagas disease		

	24h	awake state	sleep	24h	awake state	sleep
**Mean**	114.1±10.3	117.3±10.4	100.9±10.0	111.0±10.6	112.7±10.5	105.1±11.7
**SBP**						
**mmHg**						
**Mean**	68.9±7.6	71.3±8.1	59.2±7.5	66.9±7.0	68.3±7.2	62.2±8.1
**DBP**						
**mmHg**						

*Data expressed as mean and standard deviation; SBP: systolic blood pressure; DBP: diastolic blood pressure; ACD: acute Chagas disease*

## Discussion

Discussion Changes in autonomic nervous system are well characterized in chronic Chagas disease, with neuron loss and lesion in the parasympathetic pathway and increased sympathetic activity. Studies on patients with the indeterminate form of Chagas disease have shown a predominance of parasympathetic activity in these patients, which was correlated with autonomic dysfunction.^9^ Results of an interesting study indicated a relationship between changes in autonomic modulation and endothelial function in patients with ACD.^10^ Lesions in the central nervous system were found in anatomopathological studies in ACD patients, described as distant systemic lesions caused by ganglion cells.^11 , 12^ Impairment of the nervous system can be demonstrated in all stages of Chagas disease, and changes in the parasympathetic autonomic nervous system control have not been correlated with cardiovascular symptoms by functional tests on humans.^13^ The loss of autonomic control in chronic Chagas disease was described in a case-control study that evaluated the correlation between sympathetic innervation, changes in perfusion and abnormalities in the ventricular wall, showing that cardiac sympathetic dysfunction occurs in early stages of the disease and is associated with the worsening of autonomic dysfunction.^14^ However, studies on autonomic function in the acute phase of Chagas disease are scarce. Evidences have shown the involvement of the autonomic nervous system, especially the parasympathetic system, soon after initial infection, i.e., in the undetermined phase of Chagas disease.^15^

Physiological variations in BP have a circadian rhythm, with fluctuations over 24 hours and BP drop during sleep. This fall, detected by ABPM, normally exceeds 10% of BP in the awake state, and is observed in approximately 95% of the normotensive individuals.^16^ During sleep, there are specific changes in autonomic and endocrine functions, with reduced sympathetic activity and predominance of parasympathetic activity, leading to physiological BP fall.^17 , 18^ The observations in clinical practice indicating that many patients with ACD that underwent ABPM for any reason did not show nocturnal fall of BP motivated the development of a systematic study to analyze the behavior of BP in ABPM. In the control group, we included only individuals with good cardiovascular health, with no history of hypertension, diabetes or cardiovascular disease. All individuals had normal office BP (mean of the last two measures <140/90 mmHg). The only drug taken by the patients with ACD was benznidazole, an antiparasitic medication used in the treatment of *T. cruzi* infection, the causative agent of Chagas disease. Lack of BP fall during sleep was seen in a large proportion (more than half) of patients with ACD, and nocturnal increase of BP occurred in a significant proportion of patients (12.9% in SBP and 18.5% in DPB).

The neurohumoral features of the acute phase of Chagas disease are not well known, mainly due to epidemiological characteristics and difficult diagnosis. However, due to changes in the disease profile and the increase in the number of cases of oral contamination, with greater parasite load, we have found more obvious clinical manifestations.^19^ This lack of nocturnal fall in BP in the acute phase of Chagas disease may be the result of a disturbance in the autonomic nervous system. One limitation of this study is that we did not perform an analysis of heart rate variability, since our objective was to evaluate BP behavior over 24 hours.

## Conclusions

The results of this study suggest that the ABPM can be a useful tool for early detection of autonomic changes in the acute phase of Chagas disease. Since this was a descriptive study of patients with ACD, it is not possible to understand the real meaning of these changes, since there is no consensus about the reproducibility of this result and clinical outcomes at long term.

**Table 2:** – Number of patients with changes in ambulatory blood pressure monitoring (ABPM) in patients with acute Chagas disease and controls

	Controls			Acute Chagas disease		

	Women (n=30)	Men (n=24)	Total (n=54)	Women (n=30)	Men (n=24)	Total (n=54)
**Absence of nocturnal fall in SBP**	5	4	9 (16.6%)	20	20	40 (74.0%)*
**Absence of nocturnal fall in DBP**	4	3	7 (12.9%)	9	20	29 (53.7%)*
**Absence of nocturnal fall in SBP and DBP**	4	3	7 (12.9%)	16	12	28 (51.8%)*
**Nocturnal increase in SBP**	0	1	1 (1.8%)	5	2	7 (12.9%)*
**Nocturnal increase in DBP**	0	1	1 (1.8%)	5	5	10 (18.5%)*

*Data expressed as absolute and relative numbers. Chi-square test for comparisons between acute Chagas disease and control groups; p<0.05.*
